# Oocyte phenotype, genetic diagnosis, and clinical outcome in case of patients with oocyte maturation arrest

**DOI:** 10.3389/fendo.2022.1016563

**Published:** 2022-11-10

**Authors:** Lixia Zhu, Qiyu Yang, Huizi Jin, Juepu Zhou, Meng Wang, Liu Yang, Zhou Li, Kun Qian, Lei Jin

**Affiliations:** Reproductive Medicine Center, Tongji Hospital, Tongji Medical College, Huazhong University of Science and Technology, Wuhan, China

**Keywords:** oocyte maturation arrest, IVF failure, IVM, gene mutations, clinical outcome

## Abstract

**Background:**

oocyte maturation arrest (OMA) is currently one of the major causes of *in vitro* fertilization (IVF) failure, and several gene mutations were found to be associated with OMA. The purpose of this study was to identify the oocyte phenotype, genetic diagnosis, and clinical outcomes of patients with OMA and explore their possible interrelationships, thus providing a more individualized and efficient treatment strategy guidance accordingly.

**Methods:**

A retrospective study was conducted, involving 28 infertile women with OMA in the Reproductive Medicine Center of Tongji Hospital from 2018 to 2021. Whole-exome sequencing was performed for the detection of gene mutations. Patients were classified into three groups based on their oocyte phenotype, and for each group, the immature oocytes were cultured *in vitro* and mature oocytes were fertilized to evaluate both the maturation capacity and developmental potential. The clinical outcomes of OMA patients with different gene mutations or from different groups were further analyzed and compared.

**Results:**

Twenty-eight women with OMA were evaluated in this study. According to the stage of OMA, 14 (50.0%) women were classified as OMA Type-1 (GV arrest), 5 (17.9%) were OMA Type-2 (MI arrest), and 9 (32.1%) were OMA Type-3 (with both GV and MI arrest). Immature oocytes from OMA patients exhibited significantly lower maturation rates even after IVM, compared to those in general patients. Seven patients (25.0%) were detected to have deleterious variations in two genes (PATL2 and TUBB8), known to be associated with the OMA phenotype. Patients with identified mutations were found to have little opportunity to obtain offspring with their own oocytes. Among the patients without mutations identified, those classified as OMA Type-1 or Type-3 still had a chance to obtain offspring through IVF or natural pregnancy, while all patients in the Type-2 group failed to obtain live birth.

**Conclusions:**

Three different phenotypes were observed in patients with OMA. The clinical outcomes of patients were associated with the presence of gene mutations and the classification of oocyte phenotype, thus a reasonable triage system was proposed to optimize the allocation of health care resources and maximize patient benefit.

## Introduction

The earliest *in vitro* fertilization (IVF) procedure dated back to 1978. Over the last 40 years, with the wide application of assisted reproductive technology (ART) in the world, countless infertile couples have obtained newborns through IVF or intracytoplasmic sperm injection (ICSI) (1). However, there are still many special patients who cannot obtain pregnancy through ART. In 1990, Rudak et al. first described a case of idiopathic oocyte maturation disorder in IVF cycle (2). Since then, an increasing number of studies have reported a similar situation, where women undergoing IVF/ICSI could obtain a sufficient number of oocytes, yet some or even most of them were arrested at the germinal vesicle (GV) or metaphase I (MI) stage. During the IVF/ICSI cycle obtaining both mature and immature oocytes, the subsequent development of mature oocytes was negatively correlated with the proportion of maturation-arrested oocytes. When the percentage of immature oocytes exceeded 25%, the subsequent fertilization rate, blastocyst rate, and clinical pregnancy rate of mature oocytes were significantly reduced (3); when the percentage exceeded 40%, the nuclear maturation of oocytes might be abnormal and clinical pregnancy would be quite difficult (4). In some severe but extremely rare cases, a syndrome of repeated oocyte maturation failure was identified as there was repeated production of a majority of immature oocytes (5).

In recent years, scientists have been exploring the specific mechanisms of oocyte maturation arrest (OMA) (6) and have identified several related genes, including TUBB8 (7, 8), PATL2 (9, 10), TRIP13 (11), and TBPL2 (12, 13). Mutations of these genes could cause OMA at either GV or MI stage. In 2016, mutations in TUBB8 were identified as responsible for oocyte MI arrest (7). In 2017, mutations in PATL2 were firstly identified to be associated with oocyte GV arrest and a recessive inheritance pattern was detected (14). In 2020, TRIP13 was also found to be responsible for oocyte MI arrest (11), and TBPL2 mutations were found to be associated with OMA and fertilization disorders in 2021 (13). These findings are of great significance to better understand the mechanism of OMA and the relationship between gene mutations and female infertility.

However, the previous studies mainly focused on various molecular markers and the phenotypes corresponding to the gene mutations. While patients need to understand the causes of their infertility, the treatment options and prognosis are more of a subject of their concerns. Whether varied genetic mutations and OMA types will lead to different clinical outcomes is not clear so far, and personalized diagnosis and treatment processes for different OMA types also need to be established.

Therefore, the purpose of the current study is to investigate the potential association between oocyte phenotype, genetic diagnosis, and clinical outcomes of patients with OMA, by analyzing the relationship between the different OMA classifications, results of whole-exome sequencing (WES), and pregnancy chance, thus supporting the reproductive professionals to provide a more individualized fertility counseling and treatment guidance for OMA patients.

## Materials and methods

### Study population

Infertile couples undergoing IVF/ICSI in our center from January 2018 to December 2021 were reviewed and patients with abnormal oocyte maturity and WES detection results were enrolled. The detailed inclusion criteria were as follows: (a) primary infertility; (b) normal karyotype (46, XX); (c) with a sufficient number of oocytes achieved each cycle (at least 6 oocytes); (d) abnormal oocyte maturation at the day of oocyte retrieval (>50% of oocytes were immature); (e) WES was performed and identified mutations were verified by Sanger sequencing. The exclusion criteria included (a) patients with all oocytes having abnormal morphology, including abnormal zona pellucida; (b) PGT cycles; (c) cycles with missing IVF data; (d) OMA could be rescued after modified ovarian stimulation in subsequent IVF cycles.

The original study was approved by the Clinical Hospital Institutional Review Board (#[2019]S964). Each of the patients had given written informed consent before the cycle start for the data collection and the donation of their immature oocytes and sperms for research use.

### Whole-exome sequencing and mutation analysis

The WES was performed using DNA samples from the peripheral blood of patients and their family members. Genomic DNA libraries were prepared using the Agilent Human SureSelect all Exon V6 kit and exome sequencing was performed on the Illumina NovaSeq 6000 platform. Clean sequencing was compared to the human reference sequence (Hg19). Sequence variants include single nucleotide variants (SNV) and small insertions or deletions (INDel). Mutations identified by WES were verified by Sanger sequencing in patients and relatives. The Exome Aggregation Consortium (ExAC) and 1000 Genomes were used to figure out the frequency of the mutations. Sorting Intolerant From Tolerant (SIFT, http://sift.bii.astar.edu.sg/), Mutation Taster (http://www.mutaiontaster.org/), Polymorphism Phenotyping (Polyphen-2, http://genetics.bwh.harvard.edu/pph2/), and Mendelian Clinically Applicable Pathogenicity (M-CAP, http://bejerano.stanford.edu/MCAP) were applied to assess the effects of these mutations.

### Controlled ovarian hyperstimulation and oocytes retrieval

The COH protocols included gonadotropin-releasing hormone (GnRH) agonist protocol, GnRH antagonist protocol, mild stimulation protocol, and luteal phase stimulation protocol. The detailed protocols were as previously described (15). When two to three leading follicles reached a mean diameter of 18 mm, an injection of recombinant human chorionic gonadotropin (hCG) (Ovidrel; Merck-Serono) was performed. Cumulus-oocyte complexes (COCs) were retrieved by guided transvaginal ultrasound 36-38 h after hCG trigger, and cultured for subsequent IVF/ICSI according to a predetermined protocol.

### Oocyte and patients phenotype classification

The phenotypes of oocytes were observed under a light microscope to assess their morphology and maturity. Oocytes with intact germinal vesicle were defined as in GV stage, oocytes without GV or polar body were defined as in MI stage, and oocytes with a first polar body (PB1) were in MII stage. Oocytes in the GV and MI stages were defined as immature (16).

Oocyte maturation failure was defined as bad egg syndrome and its subtypes were mentioned in a previous study (5), which focused on oocytes instead of patients. An Hatirnaz and Dahan classification standard has been proposed for patients, which, however, merely applied to patients in whom all oocytes were immature (17). Considering that patients often obtained both mature and immature oocytes, even after modified ovarian stimulation in subsequent IVF cycles, and there were no appropriate classification criteria for them, we proposed a modified classification standard based on the existing one to classify patients with different OMA phenotype. The enrolled 28 women were classified as OMA Type1, Type 2, and Type 3, according to the maturity of the oocytes obtained in all cycles of the patient. In this cohort, 14 patients were classified as OMA Type-1 (GV arrest group: GV oocytes account for more than half of the oocytes retrieved), 5 were OMA Type-2 (MI arrest group: MI oocytes account for more than half of the oocytes retrieved), 9 were OMA Type-3 (Mixed arrest group: the proportion of GV or MI oocytes did not reach 50%, but the sum of the two exceeded half of the total oocyte number).

### *In vitro* maturation and evaluation of maturation capacity

After denudation, the maturity of obtained oocytes was assessed. When the proportion of immature oocytes exceeded 50%, they were collected and cultured *in vitro* in G1-plus medium (Vitrolife, Sweden) for rescue-IVM. The maturity was recorded after 24 h, and the oocytes matured within 24 h were inseminated by ICSI using the sperm of the husband of the corresponding patient, as the patients’ requirements, to increase the available embryos.

### Embryos culture and evaluation

Either by IVF or ICSI, the pronucleus was detected at 16-18 h after insemination. subsequently, the normally fertilized zygotes were cultured in G1-plus medium until day 3 for cleavage-stage embryos, and then one to two high-quality embryos were selected for fresh transfer. The surplus available embryos were frozen on day 3 or further cultured in G2-plus culture medium (Vitrolife, Sweden) to day 5 or 6 for cryopreservation. Transfer with cryopreserved embryos was performed after priming the uterus with estrogen. Specifically, when all the oocytes were found to be immature and could not mature during rescue-IVM, the corresponding cycle would be canceled.

The number of fertilized oocytes and available embryos were recorded in every individual. Successful fertilization was defined as the presence of two pronuclei (2PN), and the available embryos referred to those available for transfer, freezing, and extended culture.

### Clinical outcomes follow up

IVF/ICSI cycles related data were obtained from the electronic medical record system, and women who did not obtain a live birth during IVF/ICSI cycles were also followed up by telephone or mail. For evaluating clinical outcomes, the live birth was considered the most critical indicator. The clinical outcomes were divided into three types: (a) live birth, which referred to that the patient successfully obtained a live birth with her own oocytes; (b) still trying, which referred to that no live birth was obtained by the end of follow-up, but the patients were still striving for a new attempt; and (c) abandoning, which referred to the patient abandoning further attempts with her own oocytes and choosing adoption, oocyte donation, or just giving up.

### Statistical analyses

All data were analyzed using the Statistical Package for the Social Sciences (SPSS 22.0, IBM, Armonk, NY, United States). Categorical data were presented as the number of cases and frequency (percentage), with a Chi-Square test to assess between-group differences. Wald *P*-values were two-sided; *P*<0.05 was considered to be statistically significant.

## Results

### Clinical characteristics and oocyte phenotype

To sum up, 28 patients identified as OMA with WES results were enrolled, among which 14 were classified into OMA Type-1 as GV arrest group, 5 were OMA Type-2 as M1 arrest group, and 9 were OMA Type-3 with mixed oocyte arrest. A total of 67 IVF/ICSI cycles were involved among the 28 patients. The clinical characteristics of every patient and general information of their oocytes retrieved in previous IVF/ICSI cycles were presented in **Table 1**. The age of these patients ranged from 25 to 39 years, and the infertility duration ranged from 1 to 15 years. All patients had experienced repeated failed IVF cycles due to OMA, except four patients with identified gene mutation and one patient with unexplained infertility. Although an adequate number of oocytes could be obtained in these patients, mature oocytes accounted for less than 50%, and the numbers of available embryos were even more limited. Only 6 patients in the OMA Type-1 group (42.9%), 1 in the OMA Type-2 group (20.0%), and 3 in the OMA Type-3 group (33.3%) got available embryos during the IVF/ICSI cycles.

**Table 1:** Clinical characteristics of involved individuals and oocyte phenotype.

Group	Individuals	Age, y	Infertility duration, y	IVF/ICSI cycles, n	Oocytes retrieved, n	GV, n	MI, n	MII, n	MA, n	DA, n	Fertilized oocytes, n	Available embryos, n	Gene mutations
OMA Type-1	OMA1-1	39	15	2	71	62	2	0	4	3	0	0	PATL2
	OMA1-2	30	2	1	18	16	0	0	0	2	0	0	None
	OMA1-3	25	2	4	60	40	3	14	1	2	11	2	None
	OMA1-4	32	3	2	27	25	0	2	0	0	0	0	None
	OMA1-5	36	1	4	38	24	3	10	0	1	8	7	None
	OMA1-6	30	4	2	16	16	0	0	0	0	0	0	None
	OMA1-7	32	2	3	52	49	0	2	0	1	2	2	None
	OMA1-8	31	4	2	13	9	0	0	0	4	0	0	None
	OMA1-9	28	2	3	31	25	2	4	0	0	3	2	None
	OMA1-10	29	2	3	50	32	3	10	0	5	8	5	None
	OMA1-11	29	3	4	65	51	2	8	0	4	6	4	None
	OMA1-12	30	2	1	7	2	0	0	5^a^	0	0	0	PATL2
	OMA1-13	29	5	3	54	35	12	4	1	2	6	0	None
	OMA1-14	29	7	2	25	23	1	0	0	1	0	0	None
OMA Type-2	OMA2-1	25	6	3	29	1	20	8	0	0	1	0	None
	OMA2-2	27	2	2	36	1	21	14	0	0	0	0	None
	OMA2-3	30	2	3	24	1	20	3	0	0	1	1	TUBB8
	OMA2-4	30	5	1	11	0	9	1	0	1	0	0	TUBB8
	OMA2-5	32	2	1	13	0	13	0	0	0	0	0	TUBB8
OMA Type-3	OMA3-1	33	5	3	41	7	13	20	0	1^a^	4	0	None
	OMA3-2	37	13	3	18	8	8	0	2	0	0	0	PATL2
	OMA3-3	29	1	2	22	9	5	8	0	0	2	1	None
	OMA3-4	31	9	2	42	17	3	20	2^a^	0	5	3	None
	OMA3-5	28	7	2	28	8	9	10	0	1	4	3	None
	OMA3-6	35	6	2	28	10	7	9	0	2	1	0	None
	OMA3-7	31	6	3	27	12	11	0	4	0	0	0	None
	OMA3-8	34	2	3	36	7	13	15	0	1	5	0	None
	OMA3-9	30	4	1	22	7	2	10	2^a^	1^a^	3	0	PATL2

GV, germinal vesicle; MI, metaphase I; MII, metaphase II; MA, morphology abnormalities; DA, damaged.

a abnormal GV oocytes.

### Genetic diagnosis

By WES, 7 (25.0%) out of 28 patients were identified with mutations in two genes known to be associated with the OMA phenotype, all of which were further confirmed by Sanger sequencing. As shown in **Table 1** and **Supplemental Table 1**, two PATL2 homozygous missense mutations were identified in three patients, one mutation (c.1528C>T p.P510T) in two patients (OMA1-1 and OMA3-2) and the other (c.1376C>A p.S459Y) in OMA1-12. Two PATL2 splicing mutations (c.877-1G>A p.? and c.223-14_223-2del p.Arg75Valfs*21) were identified in one patient (OMA3-9). All the exon mutations in PATL2 showed a recessive inheritance pattern and were assessed to be harmful. The specific information about the mutations was presented in our previous publication (18). Three TUBB8 heterozygous mutations were identified in three cases: one (c.1302_1304dup p.Glu434dup) in OMA2-3, one (c.527C>T p.S176L) in OMA2-4, and the other (c.874C>A p.Q292K) in OMA2-5. All the mutations in TUBB8 showed a dominant inheritance pattern. The frequency of (c.1302_1304dup p.Glu434dup) in the population was 6.03×10^-5^. As for the other two mutations, the SIFT and Polyphen-2 predictions indicated their deleterious effects.

### IVM outcomes

Generally, immature oocytes have the competency to spontaneously mature *in vitro*. As shown in **Supplemental Figure 1**, normal GV-stage oocytes underwent GVBD within 24 h during IVM, and the PB1 was expelled within 48 h; normal MI-phase oocytes expelled PB1 within 24 h of *in vitro* culture. On the contrary, the spontaneous maturation competencies of the immature oocytes from OMA patients were dramatically impaired in extended culture for 24 h and 48 h, monitored by the Time-lapse system.

To quantify the maturation competency, the 24-h maturation rates of the immature oocytes from 55 cycles of the enrolled patients were evaluated and compared with those of the immature oocytes collected from regular ICSI cycles of patients without OMA, which were reported previously (19). As shown in **Table 2**, 56.7% of GV oocytes in the control group reached maturation within 24 h during IVM, while the GV oocytes from OMA patients presented a significantly lower maturation rate, regardless of the different OMA types (11.3%, 0.0%, and 6.0%, respectively). Similarly, while the maturation rate of MI oocytes in the control group reached 92.0% at 24 h of culture, the MI oocytes from OMA patients also showed lower maturation rates (21.4%, 7.5%, and 27.5%, respectively).

**Table 2:** Comparison of 24-h maturation rates between oocytes from patients with and without OMA.

OMA type	Cycles, n	Oocyte maturity before culture			24-h maturation rate of GV oocytes, n (%)				24-h maturation rate of MI oocytes, n (%)			
		GV, n	MI, n	MII, n	OMA group	Control group	*P* value	RR	OMA group	Control group	*P* value	RR
OMA-1	34	382	28	47	43 (11.3)	89/157 (56.7)	<0.001	0.199	6 (21.4)	69/75 (92.0)	<0.001	0.233
OMA-2	8	1	67	15	0 (0.0)		NA	NA	5 (7.5)		<0.001	0.081
OMA-3	13	50	51	75	3 (6.0)		<0.001	0.106	14 (27.5)		<0.001	0.298

### Clinical outcomes

As shown in **Table 3**, all the 7 OMA patients with gene mutations got no live birth, regardless of the different arrest stage. On the contrary, those without gene mutations seemingly had a chance to achieve pregnancy, even in a natural state. Among them, 4 out of 12 patients in GV arrest group and 2 out of 7 patients in mixed arrest group obtained live births, while the 2 patients from MI arrest group failed to get pregnant. No birth defects were reported.

**Table 3:** Clinical outcomes of the OMA patients.

OMA type	Gene mutation	Live birth	Still trying	Abandoning	Lost to follow-up
OMA-1 (n=14)	Y (n=2)	0	0	2	0
	N (n=12)	4 (1 was natural pregnancy)	2	5	1
OMA-2 (n-5)	Y (n=3)	0	0	3	0
	N (n=2)	0	1	1	0
OMA-3 (n=9)	Y (n=2)	0	0	2	0
	N (n=7)	2 (1 was natural pregnancy)	2	3	0

Y: with gene mutation; N: without gene mutation.

## Discussion

In the current study, the oocyte phenotype, genetic diagnosis, and clinical outcomes of 28 patients with OMA were comprehensively observed and analyzed to investigate their association. The results indicated that patients with OMA not only suffered constant failures in traditional IVF/ICSI cycles but also showed poor responses during rescue-IVM. Gene mutations could account for a few OMA cases and seemingly resulted in a worse prognosis (no live birth). For the patients without gene mutations, the oocyte phenotype, or oocyte arrest stage, may affect their clinical outcomes to some extent.

Oocyte maturation is a complex process of completing meiosis, and multiple genes or protein molecules are involved in this process, therefore, mutations or functional defects in any molecule affecting meiosis may lead to impaired oocyte maturation. Normally, human oocytes arrest in the first meiotic prophase until puberty. The luteinizing hormone (LH) surge in the menstrual cycle increases the level of phosphodiesterase 3 (PDE3) in oocytes, hydrolyzes cyclic adenosine monophosphate (cAMP), and simultaneously activates cell division cycle gene 25 (Cdc25), resulting in the activation of mature promoting factors (MPF), a dimer composed of cyclin-dependent kinase 1 (CDK1) and cyclin B, the resumption of meiosis in oocytes, and the breakdown of GV into MI phase. When Cdc25, PDE3, or Cdk1 are mutated or knocked out, the oocyte is unable to resume meiosis and stays in the GV phase (20–22). Anaphase promotes complex/cyclosome (APC/C) is a ubiquitin ligase that releases separase to separate homologous chromosomes, and the spindle assembly checkpoint (SAC) signaling pathway also plays an important role in regulating the alignment and segregation of the chromosomes (23, 24). Theoretically, any molecular event that affects the normal regulation of APC/C and SAC signaling pathways may lead to arrest of the transition from MI phase to anaphase and telophase (25, 26). Notably, when problems arise with one molecule in the protein family with high homology, its function might be compensated by other proteins in the family, with different degrees of compensation ultimately resulting in phenotypes of varying severity. This inconsistent compensation may cause oocytes from certain patient arrest at different stages, i.e., mixed arrest (27, 28).

However, the specific mechanism of OMA is still unclear. In recent years, with the application of sequencing technology in the field of reproduction, some possible pathogenic genes have been identified, revealing the genetic etiology of OMA. In 2016, Feng et al. analyzed an infertile family with MI arrest by WES and found that heterozygous missense mutations in TUBB8 were the cause of the disease (7). Tubulin beta 8 (TUBB8), specifically expressed in oocytes, was the most predominant form of the oocyte spindle β-tubulin (29). TUBB8 mutations affected the folding of the corresponding proteins, α/β-tubulin dimer formation, and the dynamics of microtubule action, making the spindle morphology disorganized, thus interfering with meiosis and resulting in oocyte maturation disorders (decreased PB1 expulsion rate) (7, 30). From another 23 similar families, six were found to have different TUBB8 mutations, based on which the probability of TUBB8 mutations was estimated to be about 30% in patients with OMA (7). In 2017, Chen et al. performed WES in a family with oocyte GV arrest and found homozygous nonsense mutations in protein associated with topoisomerase II homolog 2 (PATL2) (31). Studies in oocytes and Hela cells suggested that mutations in PATL2 promoted post-translational degradation of the protein, and the effect of PATL2 on oocyte maturation may be bidirectional - either up- or down-regulation of its expression could cause OMA (31). Besides, compound heterozygous mutations in the PATL2 gene were found in 4 out of 179 cases with diverse phenotypes, including both GV arrest and MI arrest (31). Most recently, TRIP13 mutation was newly identified to be associated with OMA in MI stage, and injection of TRIP13 cRNA into oocytes from one affected individual was proved to rescue the phenotype (11). In the current study, gene mutations were merely found in 7 out of 28 OMA patients, highlighting the necessity of continuing to explore other possible mechanisms.

Current approaches to improve the clinical outcomes of some cases with occasional production of immature oocytes include changing trigger method and timing (32, 33), delaying post-trigger oocyte retrieval time (34), and rescue-IVM (35). However, a repeated production of immature oocytes or a syndrome of OMA could not be solved by these strategies. In the current study, the IVM outcomes were significantly worse in patients with OMA (especially those classified in MI arrest group) than in the control population, which was consistent with previous studies (35, 36). The possible reason might be the intrinsic maturation defects in the oocytes from OMA patients which could not be rescued by modifying COH protocols or extended *in vitro* culture. Even though these oocytes could achieve MII stage after IVM, their qualities and developmental competencies were compromised (4). The intrinsic modulating mechanisms of OMA need to be investigated in future research, and more effective treatment modalities are required.

More importantly, before a satisfactory therapy is determined, a reasonable and efficient triage system based on the clinical outcomes of different types of OMA patients ought to be established. According to the results in our current study, live births were obtained in patients classified in OMA Type-1 (GV arrest group) and OMA Type-3 (mixed arrest group), surprisingly with some cases of natural pregnancy. We speculated that oocytes from patients in these two groups still had the chance to mature and develop properly, because GV arrest might be caused by some external factors, such as a high level of cAMP in follicular fluid. However, all 5 patients classified in OMA Type-2 (MI arrest group) failed to obtain live birth, possibly due to some irreversible intrinsic defects in the APC/C and SAC signaling pathway instead of abnormal MPF dynamics. In a word, it seemed that patients from MI arrest group suffered a worse prognosis, although a larger sample is needed to draw a more reliable conclusion.

**Figure 1:**
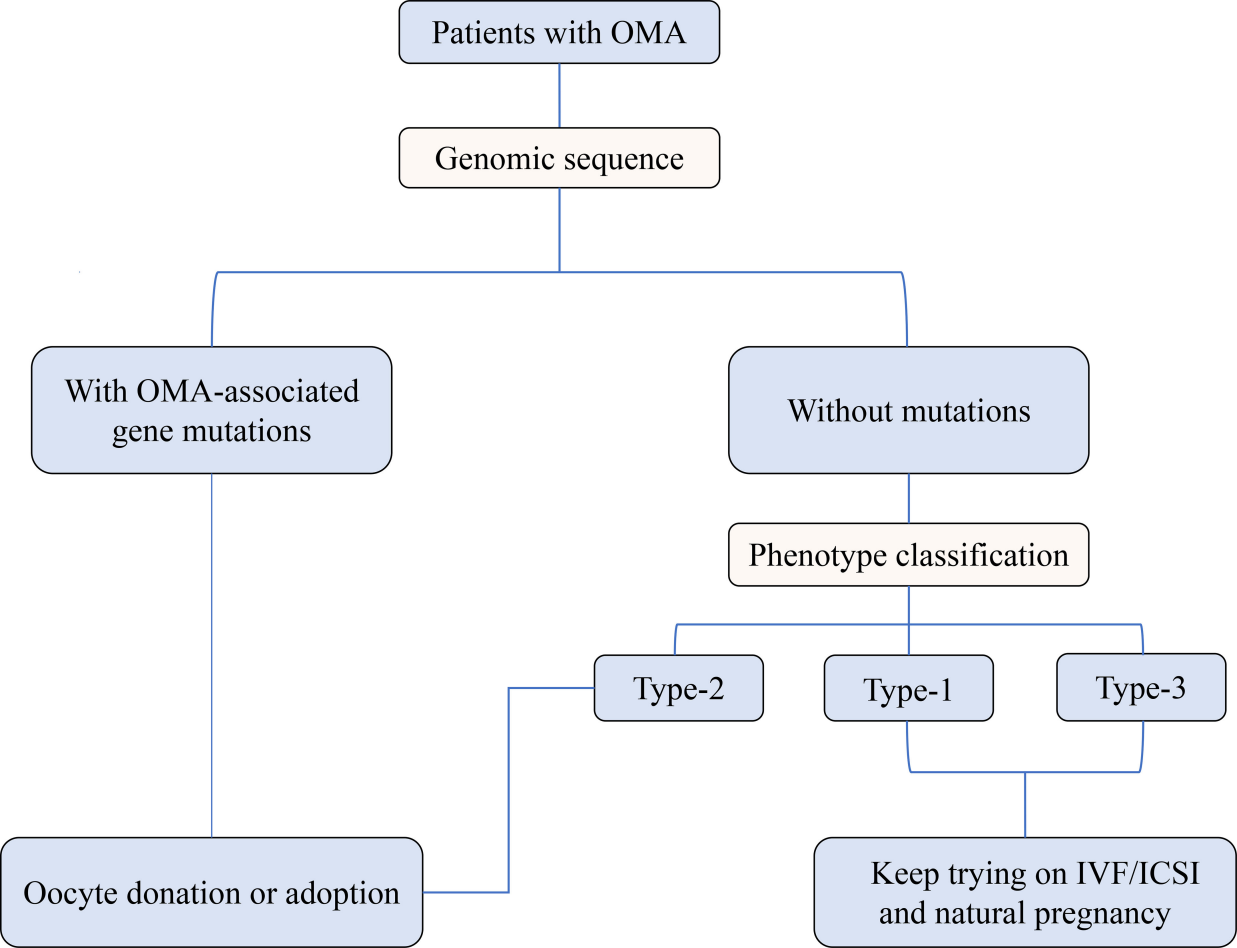
Preliminary triage system for patients with OMA based on the genetic analysis and oocyte phenotype classification.

As shown in **Figure 1**, based on the different clinical outcomes of OMA patients with or without gene mutations, the gene sequencing was recommended for those who experienced 2 to 3 failed IVF/ICSI cycles due to OMA and showed unsatisfactory IVM outcomes. Subsequently, considering the poor prognosis of OMA patients with gene mutations, we suggested that oocyte donation or adoption should be undertaken as early as possible to avoid the economic and mental burden suffered by these patients and their families during repeated failed cycles. For OMA patients without genetic mutations and classified into Type-1 or Type-3 group, who still have the possibility of conceiving through IVF/ICSI or even natural pregnancy, continued ART therapy was recommended, combined with consideration of time and cost expense. Besides, in the absence of other infertility factors, these patients should never give up trying spontaneous pregnancy. On the contrary, for patients in Type-2 group, we were reluctant and cautious to advise them to keep trying ART treatment, given that little pregnancy opportunity was observed in our study.

Based on this preliminarily proposed triage system, the treatment for the OMA patients would be more individualized and more efficient, and the allocation of health care resources would be optimized. Our first description of the relationship between genotype, oocyte phenotype, and clinical outcome in patients with OMA also laid a foundation for further targeted research. Limitations of this study include its retrospective and single-center nature, with rather small sample size. Patients are relatively heterogeneous in their clinical characteristics, such as age and infertility duration, but this also reflects the possibility of OMA affecting different populations. Besides, for patients still trying on IVF attempts, the final outcomes need to be further followed up.

## Conclusion

Three different phenotypes were observed in patients with OMA. The clinical outcomes of patients were associated with the presence of gene mutations and the classification of oocyte phenotype. The OMA patients with gene mutations were suggested to choose oocyte donation or adoption, while those without gene mutations and classified in GV arrest group or mixed arrest group could continue the ART treatment with their own oocytes, or even try spontaneous pregnancy. For those classified in MI arrest group, further IVF/ICSI should be attempted with a lot of caution for false hope and more investigations on clinical outcomes are needed.

## Data availability statement

The raw data supporting the conclusions of this article will be made available by the authors, without undue reservation.

## Ethics statement

The original study was approved by the Clinical Hospital Institutional Review Board (#[2019]S964). The patients/participants provided their written informed consent to participate in this study. Written informed consent was obtained from the individual(s) for the publication of any potentially identifiable images or data included in this article.

## Author contributions

LZ designed the study and revised the article. QY wrote the draft manuscript. HJ assisted with data collection and statistical analyses. JZ, MW, LY, ZL, KQ, LJ provided expert opinion. All authors contributed to the article and approved the submitted version.

## Funding

This work was supported by the research grants from Health Commission of Hubei Province scientific research project (WJ2021M110) and National Key Research and Development Project (2018YFC1002103).

## Conflict of interest

The authors declare that the research was conducted in the absence of any commercial or financial relationships that could be construed as a potential conflict of interest.

## Publisher’s note

All claims expressed in this article are solely those of the authors and do not necessarily represent those of their affiliated organizations, or those of the publisher, the editors and the reviewers. Any product that may be evaluated in this article, or claim that may be made by its manufacturer, is not guaranteed or endorsed by the publisher.

## Supplementary material

Supplementary Figure 1 IVM outcomes of human oocytes with or without OMA. Images of IVM process of **(A)** GV oocyte from patients without OMA; **(B)** GV oocyte from patients in GV arrest group; **(C)** MI oocyte from patients without OMA; **(D)** MI oocyte from patients in MI arrest group.

## Abbreviations

APC/C, anaphase promotes complex/cyclosome; ART, assisted reproductive technology; cAMP, cyclic adenosine monophosphate; Cdc25, cell division cycle gene 25; CDK1, cyclin-dependent kinase 1; COC, cumulus-oocyte complex; FSH, follicle-stimulating hormone; GnRH, gonadotropin-releasing hormone; GV, germinal vesicle; GVBD, germinal vesicle breakdown; hCG, human chorionic gonadotropin; ICSI, intracytoplasmic sperm injection; IVF, *in vitro* fertilization; IVM, *in vitro* maturation; LH, luteinizing hormone; MI, metaphase I; MPF, mature promoting factor; OMA, oocyte maturation arrest; PATL2, protein associated with topoisomerase II homolog 2; PDE3, phosphodiesterase 3; SAC, spindle assembly checkpoint; TUBB8, tubulin beta 8; WES, whole-exome sequencing.

## References

[1] Inhorn MC Patrizio P . Infertility around the globe: new thinking on gender, reproductive technologies and global movements in the 21st century. Hum Reprod Update (2015) 21:411–26. doi: 10.1093/humupd/dmv016 25801630

[2] Rudak E Dor J Kimchi M Goldman B Levran D Mashiach S . Anomalies of human oocytes from infertile women undergoing treatment by *in vitro* fertilization. Fertil Steril (1990) 54:292–6. doi: 10.1016/S0015-0282(16)53706-6 2379628

[3] Bar-Ami S Zlotkin E Brandes JM Itskovitz-Eldor J . Failure of meiotic competence in human oocytes. Biol Reprod (1994) 50:1100–7. doi: 10.1095/biolreprod50.5.1100 8025167

[4] Lu Y Ferrer-Buitrago M Popovic M Neupane J De Vos WH Lierman S . Patients with a high proportion of immature and meiotically resistant oocytes experience defective nuclear oocyte maturation patterns and impaired pregnancy outcomes. Reprod BioMed Online (2018) 36:396–407. doi: 10.1016/j.rbmo.2017.12.021 29609767

[5] Beall S Brenner C Segars J . Oocyte maturation failure: a syndrome of bad eggs. Fertil Steril (2010) 94:2507–13. doi: 10.1016/j.fertnstert.2010.02.037 PMC2946974 20378111

[6] Mrazek M Fulka J Jr. Failure of oocyte maturation: possible mechanisms for oocyte maturation arrest. Hum Reprod (2003) 18:2249–52. doi: 10.1093/humrep/deg434 14585868

[7] Feng R Sang Q Kuang Y Sun X Yan Z Zhang S . Mutations in TUBB8 and human oocyte meiotic arrest. N Engl J Med (2016) 374:223–32. doi: 10.1056/NEJMoa1510791 PMC4767273 26789871

[8] Chen B Wang W Peng X Jiang H Zhang S Li D . The comprehensive mutational and phenotypic spectrum of TUBB8 in female infertility. Eur J Hum Genet (2019) 27:300–7. doi: 10.1038/s41431-018-0283-3 PMC6336793 30297906

[9] Huang L Wang Y Lu F Jin Q Song G Ji J . Novel mutations in NLRP5 and PATL2 cause female infertility characterized by primarily oocyte maturation abnormality and consequent early embryonic arrest. J Assist Reprod Genet (2022) 39:711–8. doi: 10.1007/s10815-022-02412-4 PMC8995404 35091966

[10] Huo M Zhang Y Shi S Shi H Liu Y Zhang L . Gene spectrum and clinical traits of nine patients with oocyte maturation arrest. Front Genet (2022) 13:772143. doi: 10.3389/fgene.2022.772143 35140748 PMC8819080

[11] Zhang Z Li B Fu J Li R Diao F Li C . Bi-allelic missense pathogenic variants in TRIP13 cause female infertility characterized by oocyte maturation arrest. Am J Hum Genet (2020) 107:15–23. doi: 10.1016/j.ajhg.2020.05.001 32473092 PMC7332649

[12] Wang Y Xiang M Yu Z Hao Y Xu Q Kong S . A homozygous missense mutation in TBPL2 is associated with oocyte maturation arrest and degeneration. Clin Genet (2021) 100:324–8. doi: 10.1111/cge.13993 33966269

[13] Yang P Chen T Wu K Hou Z Zou Y Li M . A homozygous variant in TBPL2 was identified in women with oocyte maturation defects and infertility. Hum Reprod (2021) 36:2011–9. doi: 10.1093/humrep/deab094 33893736

[14] Maddirevula S Coskun S Alhassan S Elnour A Alsaif HS Ibrahim N . Female infertility caused by mutations in the oocyte-specific translational repressor PATL2. Am J Hum Genet (2017) 101:603–8. doi: 10.1016/j.ajhg.2017.08.009 PMC5630161 28965844

[15] Wang M Xi Q Yang Q Li Z Yang L Zhu L . The relationship between a novel evaluation parameter of premature luteinization and IVF outcomes. Reprod BioMed Online (2021) 42:323–31. doi: 10.1016/j.rbmo.2020.10.009 33250412

[16] Wu L Chen H Li D Song D Chen B Yan Z . Novel mutations in PA TL2: expanding the mutational spectrum and corresponding phenotypic variability associated with female infertility. J Hum Genet (2019) 64:379–85. doi: 10.1038/s10038-019-0568-6 30765866

[17] Hatirnaz S Başbuğ A Hatirnaz E Tannus S Hatirnaz K Bakay K . Can *in vitro* maturation overcome cycles with repeated oocyte maturation arrest? a classification system for maturation arrest and a cohort study. Int J Gynaecol Obstet (2021) 153:496–502. doi: 10.1002/ijgo.13490 33216990

[18] Liu Z Zhu L Wang J Luo G Xi Q Zhou X . Novel homozygous mutations in PATL2 lead to female infertility with oocyte maturation arrest. J Assist Reprod Genet (2020) 37:841–7. doi: 10.1007/s10815-020-01698-6 PMC7183019 32048119

[19] Yang Q Zhu L Wang M Huang B Li Z Hu J . Analysis of maturation dynamics and developmental competence of *in vitro* matured oocytes under time-lapse monitoring. Reprod Biol Endocrinol (2021) 19:183. doi: 10.1186/s12958-021-00868-0 34893069 PMC8662918

[20] Lincoln AJ Wickramasinghe D Stein P Schultz RM Palko ME De Miguel MP . Cdc25b phosphatase is required for resumption of meiosis during oocyte maturation. Nat Genet (2002) 30:446–9. doi: 10.1038/ng856 11912493

[21] Masciarelli S Horner K Liu C Park SH Hinckley M Hockman S . Cyclic nucleotide phosphodiesterase 3A-deficient mice as a model of female infertility. J Clin Invest (2004) 114:196–205. doi: 10.1172/JCI21804 15254586 PMC449752

[22] Frank-Vaillant M Jessus C Ozon R Maller JL Haccard O . Two distinct mechanisms control the accumulation of cyclin B1 and mos in xenopus oocytes in response to progesterone. Mol Biol Cell (1999) 10:3279–88. doi: 10.1091/mbc.10.10.3279 PMC25591 10512866

[23] Musacchio A Hardwick KG . The spindle checkpoint: structural insights into dynamic signalling. Nat Rev Mol Cell Biol (2002) 3:731–41. doi: 10.1038/nrm929 12360190

[24] Gorbsky GJ . The mitotic spindle checkpoint. Curr Biol (2001) 11:1001–4. doi: 10.1016/S0960-9822(01)00609-1 11747833

[25] Spruck CH de Miguel MP Smith AP Ryan A Stein P Schultz RM . Requirement of Cks2 for the first metaphase/anaphase transition of mammalian meiosis. Science (2003) 300:647–50. doi: 10.1126/science.1084149 12714746

[26] Wassmann K Niault T Maro B . Metaphase I arrest upon activation of the Mad2-dependent spindle checkpoint in mouse oocytes. Curr Biol (2003) 13:1596–608. doi: 10.1016/j.cub.2003.08.052 13678590

[27] Ryu KY Sinnar SA Reinholdt LG Vaccari S Hall S Garcia MA . The mouse polyubiquitin gene ubb is essential for meiotic progression. Mol Cell Biol (2008) 28:1136–46. doi: 10.1128/MCB.01566-07 PMC2223379 18070917

[28] Lipkin SM Moens PB Wang V Lenzi M Shanmugarajah D Gilgeous A . Meiotic arrest and aneuploidy in MLH3-deficient mice. Nat Genet (2002) 31:385–90. doi: 10.1038/ng931 12091911

[29] Feng R Yan Z Li B Yu M Sang Q Tian G . Mutations in TUBB8 cause a multiplicity of phenotypes in human oocytes and early embryos. J Med Genet (2016) 53:662–71. doi: 10.1136/jmedgenet-2016-103891 PMC5035199 27273344

[30] Chen B Li B Li D Yan Z Mao X Xu Y . Novel mutations and structural deletions in TUBB8: expanding mutational and phenotypic spectrum of patients with arrest in oocyte maturation, fertilization or early embryonic development. Hum Reprod (2017) 32:457–64. doi: 10.1093/humrep/dew322 27989988

[31] Chen B Zhang Z Sun X Kuang Y Mao X Wang X . Biallelic mutations in PATL2 cause female infertility characterized by oocyte maturation arrest. Am J Hum Genet (2017) 101(4):609–15. doi: 10.1016/j.ajhg.2017.08.018 PMC5630194 28965849

[32] Griffin D Feinn R Engmann L Nulsen J Budinetz T Benadiva C . Dual trigger with gonadotropin-releasing hormone agonist and standard dose human chorionic gonadotropin to improve oocyte maturity rates. Fertil Steril (2014) 102:405–9. doi: 10.1016/j.fertnstert.2014.04.028 24842671

[33] Vandekerckhove F Gerris J Vansteelandt S De Baerdemaeker A Tilleman K De Sutter P . Delaying the oocyte maturation trigger by one day leads to a higher metaphase II oocyte yield in IVF/ICSI: a randomised controlled trial. Reprod Biol Endocrinol (2014) 12:31. doi: 10.1186/1477-7827-12-31 24758641 PMC4008411

[34] Weiss A Neril R Geslevich J Lavee M Beck-Fruchter R Golan J . Lag time from ovulation trigger to oocyte aspiration and oocyte maturity in assisted reproductive technology cycles: a retrospective study. Fertil Steril (2014) 102:419–23. doi: 10.1016/j.fertnstert.2014.04.041 24880653

[35] Hourvitz A Maman E Brengauz M Machtinger R Dor J . *In vitro* maturation for patients with repeated *in vitro* fertilization failure due to ‘oocyte maturation abnormalities’. Fertil Steril (2010) 94:496–501. doi: 10.1016/j.fertnstert.2009.03.040 19589517

[36] Galvão A Segers I Smitz J Tournaye H De Vos M . *In vitro* maturation (IVM) of oocytes in patients with resistant ovary syndrome and in patients with repeated deficient oocyte maturation. J Assist Reprod Genet (2018) 35:2161–71. doi: 10.1007/s10815-018-1317-z PMC6289928 30238176

