# Supplementary material for: Oocyte phenotype, genetic diagnosis, and clinical outcome in case of patients with oocyte maturation arrest
Source: Front Endocrinol (Lausanne). 2022 Nov 10;13:1016563. doi: 10.3389/fendo.2022.1016563 (PMC9684610; doi:10.3389/fendo.2022.1016563)
Supplement: Supplementary file 1 [file Table_1.docx]

**Supplemental Table 1 Analysis of mutations identified by whole-exome sequencing**

| **Individuals** | **Chromosome** | **Gene** | **Mutation** | **Genetic Pattern** | **Exon** | **Mutation type** | **Allele frequency** | **SIFT** | **Polyphen-2** | **Mutation Taster** | **M-CAP** |
| --- | --- | --- | --- | --- | --- | --- | --- | --- | --- | --- | --- |
| OMA1-1 | chr15:44958675 | PATL2 | c.1528C>T p.P510T | AR | 15 | Missense | NA | D | P | D | D |
| OMA1-12 | chr15:44959391 | PATL2 | c.1376C>A p.S459Y | AR | 14 | Missense | NA | D | P | D | D |
| OMA2-3 | Chr10:93028-93030 | TUBB8 | c.1302_1304dup p.Glu434dup | AD | 4 | Inframe insertion | 6.03×10^-5^ | NA | NA | NA | NA |
| OMA2-4 | Chr10:93805 | TUBB8 | c.527C>T p.S176L | AD | 4 | Missense | NA | D | P | NA | Likely Benign |
| OMA2-5 | Chr10:93458 | TUBB8 | c.874C>A p.Q292K | AD | 4 | Missense | NA | D | P | NA | Likely Benign |
| OMA3-2 | Chr15:44958675 | PATL2 | c.1528C>T p.P510T | AR | 15 | Missense | NA | D | P | D | D |
| OMA3-9 | Chr15:44961762 | PATL2 | c.877-1G>A p.? | AR | intron | Splicing | 6.53×10^-6^ | NA | NA | NA | NA |
|  | Chr15:44966430 | PATL2 | c.223-14_223-2d  el p.Arg75Valfs*21 |  |  |  | 2.1×10^-4^ |  |  |  |  |

Analysis of the harmfulness of mutations by using Sorting Intolerant From Tolerant (SIFT), Polymorphism Phenotyping (Polyphen-2), Mutation Taster, and Mendelian Clinically Applicable Pathogenicity (M-CAP). AR, autosomal recessive **inheritance; AD, autosomal dominant inheritance;** NA, not applicable; D, damaging; P, probably damaging.
