# Supplementary figures and images for: Oocyte phenotype, genetic diagnosis, and clinical outcome in case of patients with oocyte maturation arrest
Source: Front Endocrinol (Lausanne). 2022 Nov 10;13:1016563. doi: 10.3389/fendo.2022.1016563 (PMC9684610; doi:10.3389/fendo.2022.1016563)

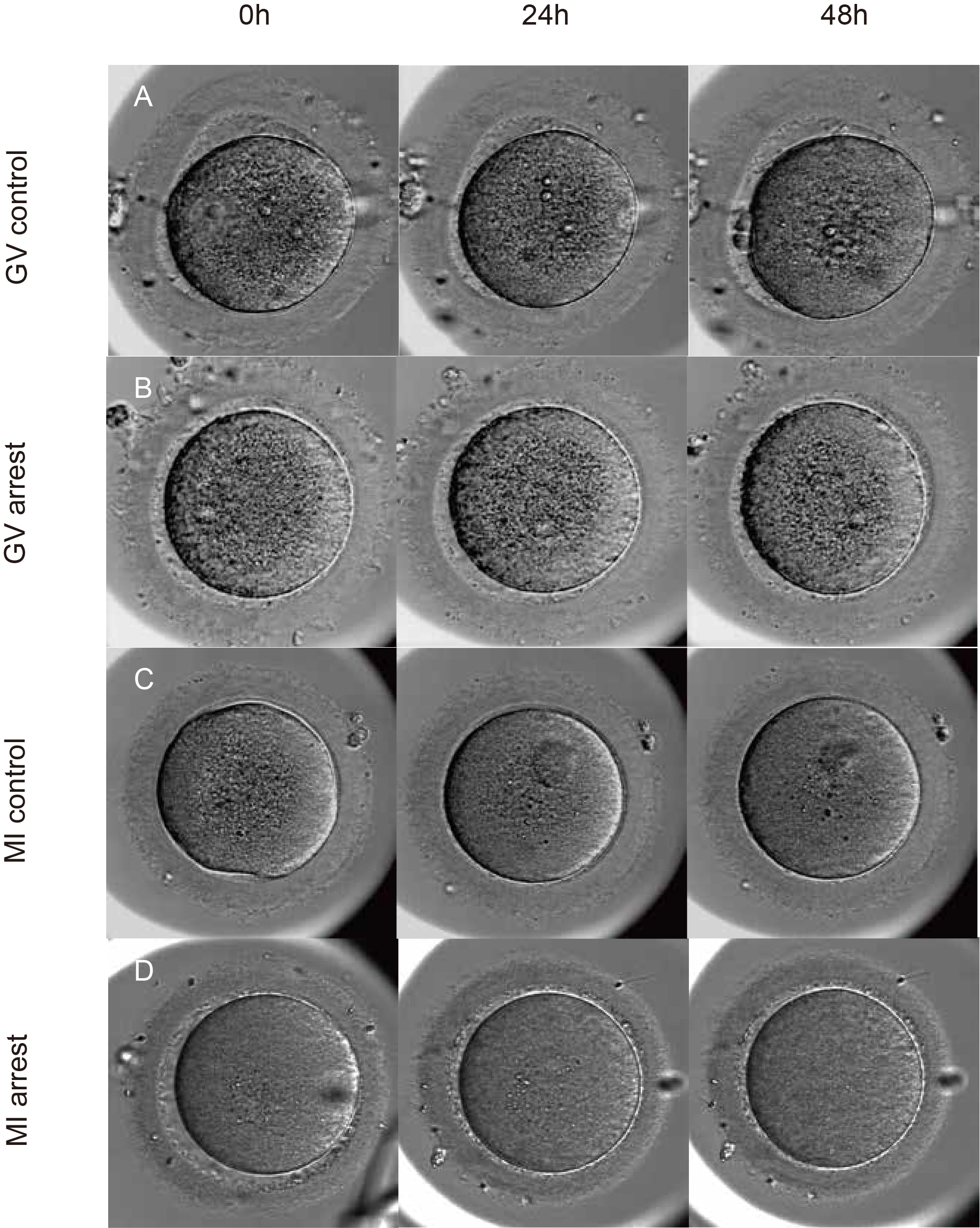

Supplement: Supplementary Figure 1 — IVM outcomes of human oocytes with or without OMA. Images of IVM process of (A) GV oocyte from patients without OMA; (B) GV oocyte from patients in GV arrest group; (C) MI oocyte from patients without OMA; (D) MI oocyte from patients in MI arrest group. [file Image_1.png]
